# Supplementary material for: Analysis of the Factors Affecting Static In Vitro Pepsinolysis of Food Proteins
Source: Molecules. 2022 Feb 14;27(4):1260. doi: 10.3390/molecules27041260 (PMC8878058; doi:10.3390/molecules27041260)
Supplement: Supplementary file 1 [file molecules-27-01260-s001.zip › Supplementary Figure S1.pdf]

Analysis of the Factors Affecting Static In Vitro Pepsinolysis of Food Proteins

Natsumi Maeda, Dorota Dulko, Adam Macierzanka and Christian Jungnickel *

Department of Colloid and Lipid Science, Faculty of Chemistry, Gdańsk University of Technology, ul. Narutowicza 11/12, 80-233 Gdańsk, Poland

* Correspondence: christian.jungnickel@pg.edu.pl; Tel.: +48-583472469

Re-calculation for protein and pepsin concentration

Among those ten categories, the concentration of protein and pepsin required recalculation. The concentration of each solution was evaluated at the state at which the protein solution was mixed with the simulated gastric fluid (SGF). We applied mainly two approaches based on given data from the papers. If possible the data was collected directly from the papers, however, if a mixture of stock solutions was presented the concentrations were recalculated accordingly.

For a couple of papers, protein mass was indicated, but precise protein composition (e.g. β-Casein, ALA, BLG) was not provided. In those instances, we adopted two methods. 1. If the composition of the protein is well known the composition from literature was averaged and used to determine a concentration of a studied protein. For example, the work by Sakai [1] proteolysis BLG of cow milk protein from infant formula was assessed. Mass of infant formula in the protein stock solution, percentage of protein of the infant formula as well as a volume of SGF are provided, allowing for the determination of the concentration of protein solution (5.2 mg/mL), yet the composition of BLG protein was not clarified. Therefore, literature values detailing cow milk protein composition were collected specifically for BLG: 9.24% [2], 9.73% [3], and 8.34% [4] resulting in an average of 9.1%, which was used for further calculations (i.e. the BLG concentration was estimated to be approximately 0.47mg/mL). 2. If the composition of the protein was not known or literature values were not found the band intensity of SDS PAGE was utilized. For this, GelAnalyzer 19.1 [5] was used on the SDS-PAGE figures. For example, Luo et al. [6], investigated the pepsinolysis of the α-subunit and the β-subunit from fish parvalbumin. The study included a concentration of protein solution at which the digestion starts (0.67mg/mL) but without the composition of α-subunit and β-subunit proteins. SDS-PAGE profiles of pepsinolysis in the paper were utilized for analyzing the intensity of the bands for each subunit, allowing for the determination of the final protein concentrations of 0.317mg/mL (α-subunit) and 0.349mg/mL (β-subunit).

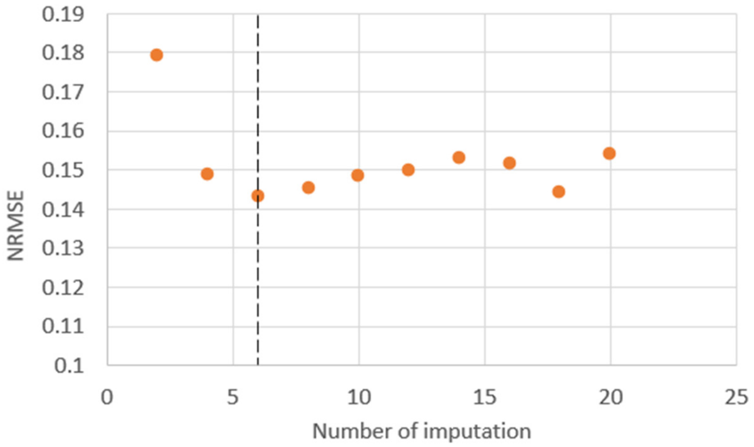

Figure S1. Determination of the optimal number of imputations. The optimal number of imputations was determined to be 6, as indicated by the dashed line. The NRMSE is the normalized root mean squared error.

References

1. Sakai, K.; Yoshino, K.; Satter, M.A.; Ota, F.; Nii, Y.; Fukuta, K.; Ueda, N.; Shimizu, Y.; Yamamoto, S. Note Effects of pH Variation and NaCl on In Vitro Digestibility of Cow's Milk Proteins in Commercially Available Infant Formulas; 2000, 46, 325–328.

2. Bonfatti, V.; Di Martino, G.; Carnier, P. Effectiveness of mid-infrared spectroscopy for the prediction of detailed protein composition and contents of protein genetic variants of individual milk of Simmental cows. *J. Dairy Sci.* **2011**, *94*, 5776–5785, doi:10.3168/jds.2011-4401.

3. Gellrich, K.; Meyer, H.H.D.; Wiedemann, S. Composition of major proteins in cow milk differing in mean protein concentration during the first 155 days of lactation and the influence of season as well as shortterm restricted feeding in early and mid-lactation. *Czech J. Anim. Sci.* **2014**, *59*, 97–106, doi:10.17221/7289-cjas.

4. Schopen, G.C.B.; Visker, M.H.P.W.; Koks, P.D.; Mullaart, E.; van Arendonk, J.A.M.; Bovenhuis, H. Whole-genome association study for milk protein composition in dairy cattle. *J. Dairy Sci.* **2011**, *94*, 3148–3158, doi:10.3168/jds.2010-4030.

5. Lazar Jr., I.; Lazar Sr., I. GelAnalyzer 19.1 2010.

6. Luo, C.; Guo, Y.; Li, Z.; Ahmed, I.; Pramod, S.N.; Gao, X.; Lv, L.; Lin, H. Lipid emulsion enhances fish allergen parvalbumin's resistance to in vitro digestion and IgG/IgE binding capacity. *Food Chem.* **2020**, *302*, 125333, doi:10.1016/j.foodchem.2019.125333.
